# Supplementary material for: A New Method for Improving Extraction Efficiency and Purity of Urine and Plasma Cell-Free DNA
Source: Diagnostics (Basel). 2021 Apr 3;11(4):650. doi: 10.3390/diagnostics11040650 (PMC8067265; doi:10.3390/diagnostics11040650)
Supplement: Supplementary file 1 [file diagnostics-11-00650-s001.pdf]

A new method for improving extraction efficiency and purity of urine and plasma cell-free DNA

Selena Y. Lin, Yue Luo, Matthew M. Marshall, Barbara J. Johnson, Sung R. Park, Zhili Wang, Ying-Hsiu Su

Supplemental Data

Figure S1. *TP53* DNA concentrations in cfDNA isolated from six normal urine donors per kit.

Figure S2. Protein contamination in cfDNA extracted from plasma (2mL) of six normal donors by three cfDNA extraction kits as measured by Qubit total protein assay.

Figure S3. *TP53* DNA concentrations isolated from six normal plasma donors per kit.

Table S1. Statistical results of Tukey's post hoc comparisons for recovery of synthetic 141 bp DNA from 3mL urine samples with kits J, M, and Q.

Table S2. Liquid biopsy donor sample information.

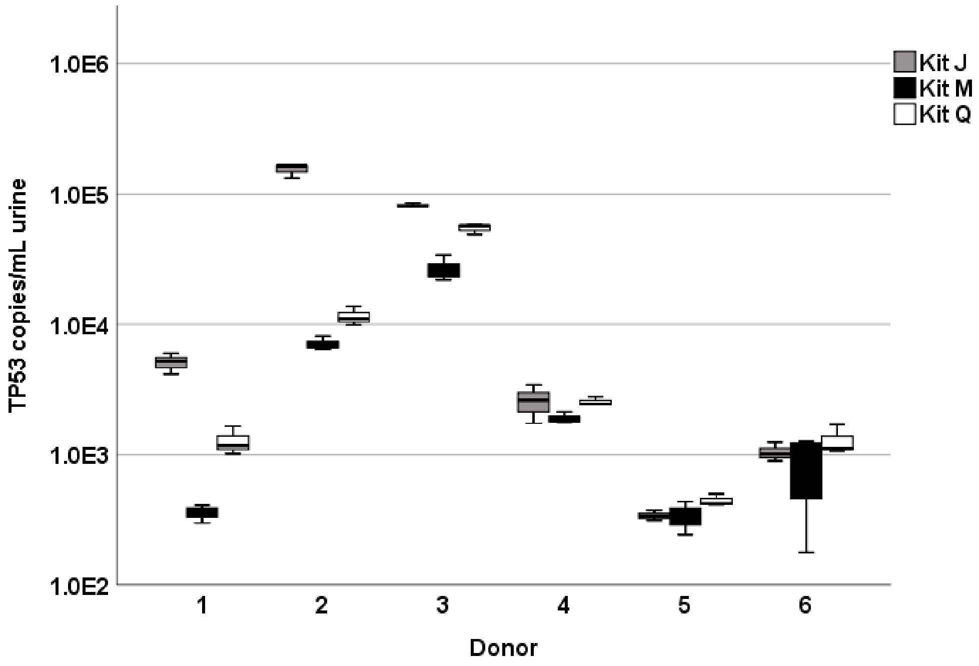

Figure S1. *TP53* DNA concentrations in cfDNA isolated from six normal urine donors per kit. Average *TP53* concentrations of three extraction replicates for each donor are shown.

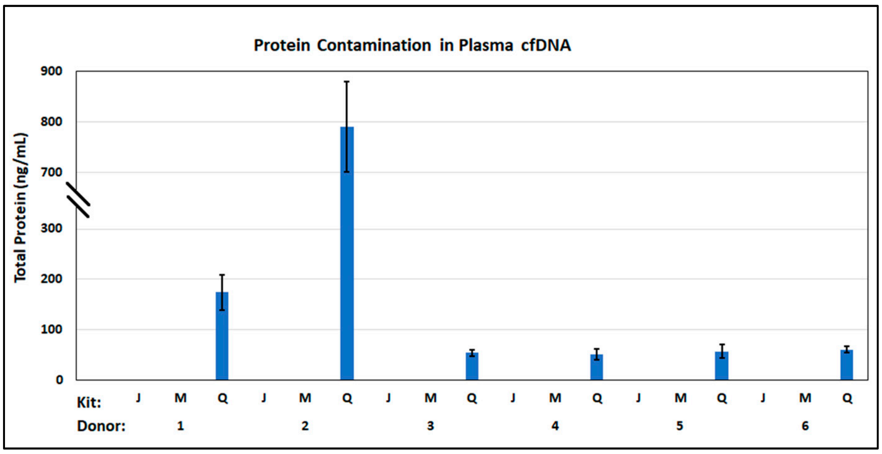

Figure S2. Protein contamination in cfDNA extracted from plasma (2mL) of six normal donors by three cfDNA extraction kits as measured by Qubit total protein assay. DNA input equivalent of 0.2mL of plasma was used.

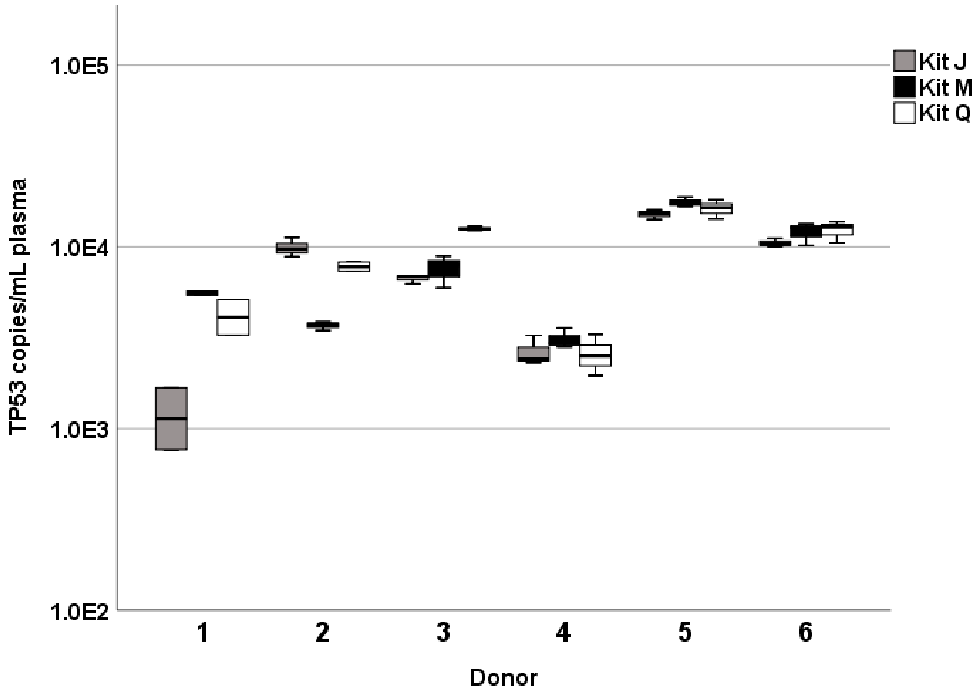

Figure S3. ***TP53*** **DNA concentrations isolated from six normal plasma donors per kit.** Average *TP53* copies/mL concentrations are shown per donor from three extraction replicates per kit.

Table S1. Statistical results of Tukey's post hoc comparisons for recovery of synthetic 141 bp DNA from 3mL urine samples with kits J, M, and Q.

Contrast	Estimate	95% CI	d.f.	t	p-value
J - M	0.75	0.63-0.86	44	11.72	<0.001
J - Q	0.65	0.51-0.79	44	9.94	<0.001
M - Q	-0.1	-0.21-0.02	44	-1.61	0.25

95% CI, 95% confidence interval; df, degrees of freedom; t, t-value.

Table S2. Liquid biopsy donor sample information.

Biopsy	Donor #	Sex	Biopsy extraction volume (mL)
Urine	1	F	3
Urine	2	F	
Urine	3	F	
Urine	4	M	
Urine	5	M	
Urine	6	M	
Plasma	1	M	2
Plasma	2	M	
Plasma	3	M	
Plasma	4	F	
Plasma	5	F	
Plasma	6	F	
PG Urine	Donor 1 T1	F	4mL
PG Urine	Donor 1 T2	F	4mL
PG Urine	Donor 2 T1	F	4mL
PG Urine	Donor 2 T2	F	4mL
PG Urine	Donor 2 T3	F	4mL
PG Plasma	Donor 1 T1	F	0.5mL
PG Plasma	Donor 1 T2	F	0.5mL
PG Plasma	Donor 1 T3	F	0.5mL
PG Plasma	Donor 1 T4	F	0.5mL
PG Plasma	Donor 1 T5	F	0.5mL
PG Plasma	Donor 3	F	0.5mL
PG Plasma	Donor 4	F	0.5mL
